# Transposition of the Great Arteries With Ventricular Septal Defect in a One-Month-Old Infant

**DOI:** 10.7759/cureus.62888

**Published:** 2024-06-22

**Authors:** Abhiram A Sahasrabhojanee, Sunil Kumar, Somya Gupta

**Affiliations:** 1 Medicine and Surgery, Jawaharlal Nehru Medical College, Datta Meghe Institute of Higher Education and Research, Wardha, IND; 2 Medicine, Jawaharlal Nehru Medical College, Datta Meghe Institute of Higher Education and Research, Wardha, IND; 3 Ophthalmology, Jawaharlal Nehru Medical College, Datta Meghe Institute of Higher Education and Research, Wardha, IND

**Keywords:** ventricles, septum, aorta, pulmonary artery, congenital, transposition

## Abstract

Transposition of the great arteries (TGA), also termed complete transposition, is a congenital cardiac defect, and it is subjected to the concordance of the atrioventricular system discordance of the ventriculoarterial (VA) system. Male babies have slightly more preponderance than female babies. In almost half of the cases reported, the discordance in the VA system is the sole finding. In 10% of cases, TGA is often caused by other cardiac deformities, which incorporate defects in the septum of the ventricular chamber and stenosis in the left ventricular outlet. Eventually, these recite the progression, prognosis, and clinical presentation. In most cases, the onset is as soon as the birth. However, it may vary and depend on the difference in the anatomical and functional types, which determine the level of amalgamation between the oxygenated and deoxygenated blood. The etiology of the following condition is still unknown, but this defect is known to have a genetic preponderance. The definitive management of this condition is surgery, but medical management is optional and is prescribed according to the clinical evaluation and includes furosemide. Surgical correction can be performed in the later course of time, ensuring the fitness of the child. The procedure of choice is the arterial switch operation. This case report emphasizes the vital function of extensive critical care of newborns and prompt antenatal screening of high-risk pregnancies. It also highlights the importance of quick healthcare practices for newborns with congenital disabilities.

## Introduction

Transposition of the great arteries (TGA) befalls when the aorta originates from the anatomical right chamber of the heart. By contrast, the pulmonary artery originates from the anatomical left chamber of the heart. Complete transposition of the great arteries is referred to as d-TGA, with the letter "d" signifying the dextroposition. The aorta is additionally encountered in the proper and anterior heart regions. There must be concurrent contact between the oxygenated and deoxygenated blood flow [1]. However, there may be concurrent defects in the septum, either in the ventricular or the atrial region. Patency of the duct binding the aorta and the pulmonary artery is present in the first week of life, which may also help sustain the entity. Typically, concomitant lesions generally presenting are VSD (occurring in about 50% of cases), pulmonary outlet stenosis, and, less typically, aortic coarctation [2]. This defect is not quite common, and the incidence is approximately one in 5,000 live births. The arterial switch operation (ASO) was first undertaken as the therapy of selection in individuals with transposition of the arteries and septal defect of the ventricles, which were thought to be the sole determinant of the structural repair for the same [3]. TGA with an underlying defect in the septum between the two ventricles and obstruction in the outlet from the left ventricle represents a small fraction of all congenital cardiac defects [4]. Obstruction in the outflow tract of the left ventricle at the level of the VSD occurs in up to 10% of patients [5]. The procedure of Rastelli was developed for the surgical care of patients associated with this fatal condition [6]. This procedure has become the preferred surgical approach for treating this lesion, but its lingering outcomes could have been better. This procedure's most frequently occurring complications include conduit blockage and palpitations [7].

## Case presentation

A one-month-old male child was brought to the tertiary care center with chief complaints of cough and bluish discoloration of palms and soles for 20 days; the mother also complained of increased work of breathing for three days. As narrated by the mother, the child was apparently healthy 20 days ago when the child developed a cough. The cough was insidious in onset; it coincided with the onset of other symptoms. The cough was dry and nonproductive. The cough did not elicit a typical response due to the infant's young age. The consistency of the cough was persistent and occurred throughout the day and night. It was exacerbated when the child changed positions, especially after attaining the supine position. The cough was not associated with post-tussive vomiting. The mother also detected that the infant's palms and soles would turn blue on excessive crying. This phenomenon was noted to occur intermittently and was particularly pronounced during periods of increased respiratory effort, such as during feeding or crying spells. It resolved shortly after the cessation of crying episodes or feeding. The mother also reported that the child further developed increased work breathing, for which they took him to a private hospital. Moreover, he was admitted and referred to the tertiary care center for further management.

There was no history or complaint of intermittent fever, ruling out any infectious etiology. Mainly, the suck-rest-suck cycle was observed to be positive. In addition, the mother reported a history of sweating on the forehead, potentially indicative of increased respiratory effort. However, a history of decreased appetite or loose stools was not reported, suggesting that the gastrointestinal system was unaffected. In addition, no indications of altered sensorium indicated the absence of any neurological manifestations. These findings give a crucial outline of the child’s clinical manifestation and help in the prompt surveillance of the infant’s illness.

According to the birth history, the child’s weight was 2.8 kilograms. It was a vaginal delivery occurring at full term. Following birth, the infant had a history of a brief stay at the Neonatal Intensive Care Unit (NICU) for a one-day duration due to hyperbilirubinemia. Management during the NICU stay was primarily focused on the treatment of hyperbilirubinemia. Apart from this brief medical intervention, the further clinical course of the infant was uneventful. There were no other complications during the neonatal period.

The past medical narrative of the case reveals pertinent facts that play a crucial function in understanding the child’s clinical journey since birth. As narrated by the mother, 15 days before the current presentation, the child had been admitted to a private hospital for a duration of five days with the suspicion of bronchopneumonia. In the subsequent tests and workup done during this admission, which included a 2D echocardiogram, it came to notice that the child had a congenital heart defect and was diagnosed with transposition of great arteries with a concurrent perimembranous type of ventricular septal defect measuring 6 mm in diameter. The child's developmental milestones were reported within expected ranges, and the mother did not report any notable delays or abnormalities. According to the national immunization schedule, the child was immunized at birth, and there was no history of any previous surgical interventions. The family history regarding congenital anomalies or other hereditary conditions was insignificant.

Physical examination

For the subsequent investigations, an extensive general and systemic examination was done to assess the child’s overall condition, particularly cardiovascular complaints. The findings of this clinical examination are summarized in Table 1. It provides a crucial insight into the infants’ physical status and serves as a footing for successive diagnostic and restoring interventions.

**Table 1:** Findings of the physical examination conducted BP: blood pressure; S1: heart sound 1; S2: heart sound 2

System	Examination findings
General appearance	Alert but irritable and lethargic due to inadequate oxygenation
Skin	Cyanosis was observed during excessive crying, particularly around lips, nail beds, and extremities.
Vitals	BP: 90/60 mmHg; heart rate: 148 beats/minute; respiratory rate: 16 cycles/minute; peripheral capillary oxygen saturation: 64%
Respiratory system	Respiratory rate: 66 cycles/ minute; breath sounds bilateral; subcostal retractions present; nasal flaring was also present; no wheezing, rales, or rhonchi
Cardiovascular system	S1 was heard with single and loud S2; pan systolic murmur was also heard the most audible along the left lower border of the sternum; apical impulse felt at the fourth intercostal space lateral to the midclavicular line; no parasternal heave; no diastolic thrill present.
Abdomen	Soft and non-tender; no distention or visible pulsations; no organomegaly or palpable masses
Neurological examination	The anterior fontanelle is wide and at level, alert and responsive, with normal tone and strength and normal reflexes.
Growth parameters	Weight, length, and head circumference within normal range

Investigation

An extensive array of tests were conducted to assess the patient’s condition. It included a blood profile with a peripheral smear. A coagulation profile was done, which is crucial before any surgical intervention. C-reactive protein (CRP), which indicates the levels of inflammation, was also done, which prompts the body’s immune and inflammatory response to the underlying cardiac defect. Enzymes of the liver and kidney levels were also done to asses any particular organ damage. A viral panel was also conducted to rule out viral infections, if any. The results of this array of tests are summarized in Table 2.

**Table 2:** Findings of the laboratory investigations LFT: liver function test; ALT: alanine transaminase; AST: aspartate transaminase; ALP: alkaline phosphatase; KFT: kidney function test; Na: sodium; K: potassium; CBC: complete blood count; Hb: hemoglobin; WBC: white blood cells; RBC: red blood cells; PT: prothrombin time; INR: international normalized ratio; APTT: activated partial thromboplastin time; MCHC: mean corpuscular hemoglobin concentration; MCV: mean corpuscular volume; HBsAg: hepatitis b surface antigen; HCV: hepatitis c virus

Test	Observed value	Reference range
CBC		
Haemoglobin	12.8 g/dL	10-15 g/dL
WBC	7.7 x 10^3^/µL	4.0-11.0 x 10^3^/µL
RBC	4.6 x 10^6^/µL	4.1-6.1 x 10^6^/µL
Platelets	280 x 10^3^/µL	150-450 x 10^3^/µL
MCHC	31.4 g/dL	32-36 g/dL
MCV	87.6 fL	80-100 fL
KFT		
Blood urea nitrogen	11 mg/dL	7-20 mg/dL
Serum creatinine	0.4 mg/dL	0.6-1.3 mg/dL
Na	137 mmol/L	135-145 mmol/L
K	5.5 mmol/L	3.5-5.0 mmol/L
LFT		
Total bilirubin	1.4 mg/dL	0.3-1.0 mg/dL
Direct bilirubin	0.1 mg/dL	0.0-0.3 mg/dL
Indirect bilirubin	1.3 mg/dL	0.1-0.7 mg/dL
Alkaline phosphatase (ALP)	(186). U/L	44-147 U/L
Aspartate aminotransferase (AST)	65 U/L	5-40 U/L
Alanine aminotransferase (ALT)	33 U/L	7-56 U/L
Albumin	3.8 g/dL	3.5-5.5 g/dL
Total protein	6.1 g/dL	6.0-8.3 g/dL
Coagulation profile		
PT	14 seconds	10.0-14.0 seconds
INR	1.17	0.8-1.2
APTT	51 seconds	25-35 seconds
Virology report		
HBsAg	Non-reactive	
HCV	Non-reactive	
C-reactive protein	0.723	Less than 1.0 mg/dL

A chest X-ray was taken from the posteroanterior (PA) view. The thorax X-ray findings show that the heart appears elongated and narrow, resembling an egg on a string due to the odd location of the great arteries. The X-ray also suggests cardiomegaly, which is the enlargement of the heart. Pulmonary vascularity is increased, resulting in prominent pulmonary vascularity on X-ray. Other structures like the diaphragm, the costophrenic angles, and the bone and soft tissues appear to be expected. These findings, significantly when correlated with clinical symptoms and other diagnostic tests like echocardiography, which is mentioned subsequently, aid in the diagnosis of transposition of great arteries.

Echocardiography

**Figure 1:**
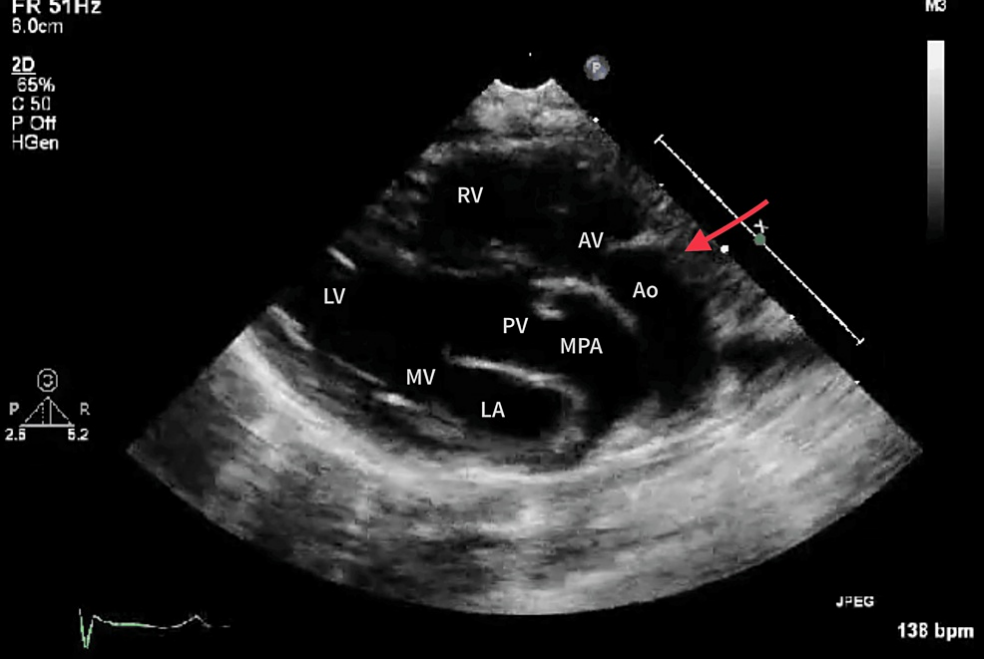
Parasternal long-axis view suggesting transposition of the great arteries Red line showing the aorta originating from the right ventricle. LV, left ventricle; RV, right ventricle; MV, mitral valve; PV, pulmonary valve; LA, left atrium; Ao, aorta; AV, aortic valve; MPA, main pulmonary artery

A thorough echocardiographic evaluation was conducted on the patient, revealing these significant findings. The diagnosis of transposition of great arteries was confirmed, accompanied by a 6 mm peri-membranous non-restrictive ventricular septal defect (VSD) with the blood flowing from the left to right direction. In addition, there was evidence of dilated and confluent pulmonary arteries, which indicate severe pulmonary artery hypertension. Regardless of these findings, the left and the right ventricular processes were within normal limits. The ejection fraction was measured to be 60%. However, a malposed left ventricle was observed. The aorta was arranged on the right side and was anterior to the pulmonary artery. The further assessment also revealed a slight arch of the aorta at the isthmus, measuring 2.4 mm, and a transverse arch diameter of 5.5 mm, but there was not any significant gradient across the arch. Figure 1 shows the parasternal long-axis view. It indicates that the aorta becomes apparent anteriorly from the right side of the heart, and the pulmonary artery becomes apparent posteriorly from the left side. Bifurcation of the pulmonary artery into its branches is seen.

**Figure 2:**
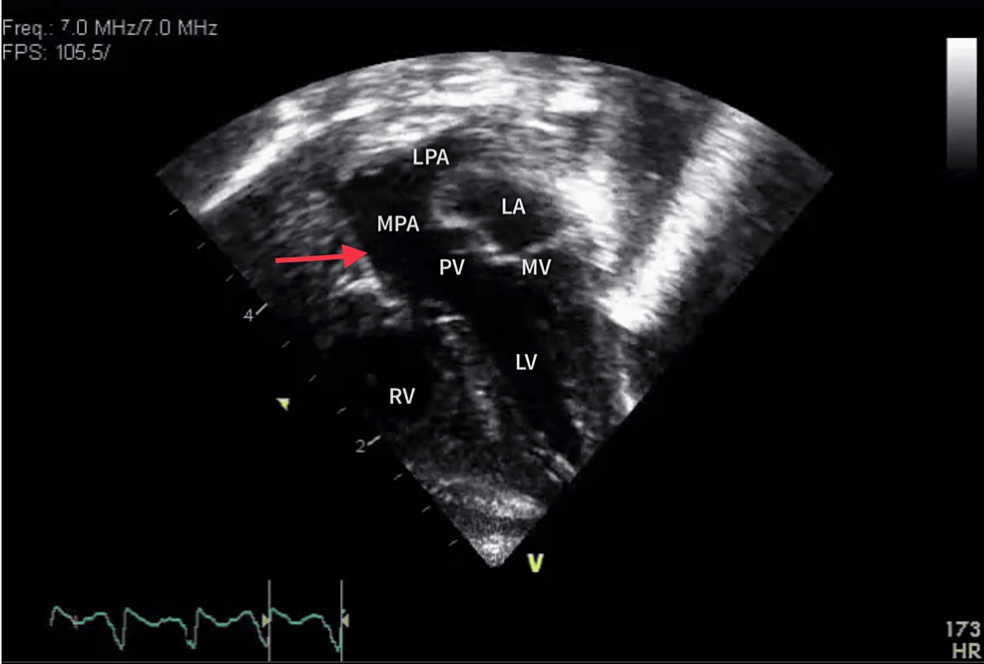
Apical five-chamber view The red arrow shows the pulmonary artery originating from the left ventricle. RV, right ventricle; LV, left ventricle; MV, mitral valve; PV, pulmonary valve; MPA, main pulmonary artery; LPA, left pulmonary artery; LA, left atrium

The apical five-chamber view corresponding to Figure 2 suggests the pulmonary artery originating from the left ventricle.

**Figure 3:**
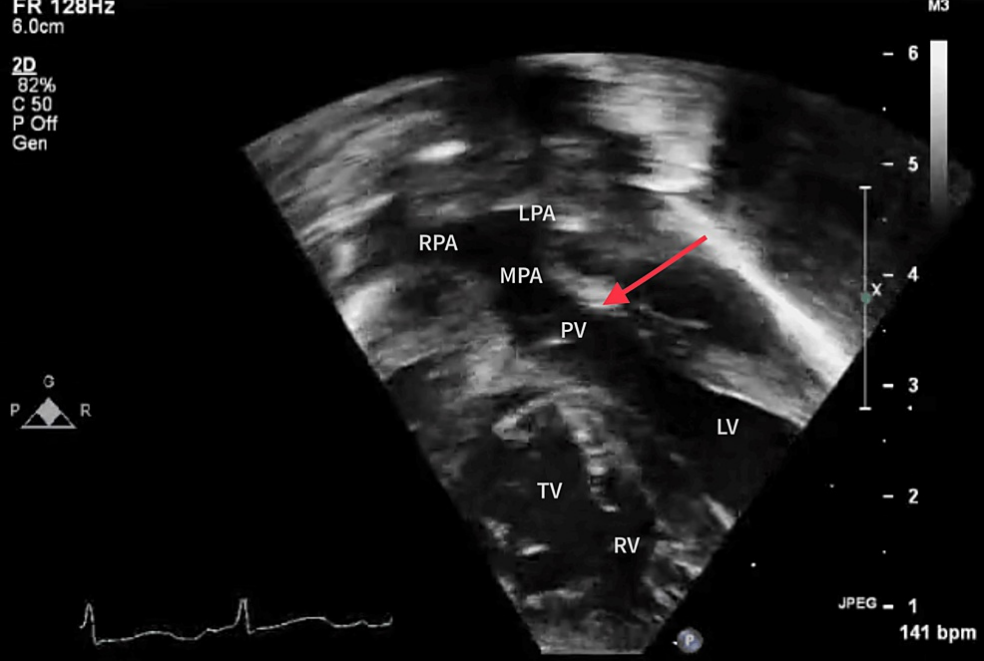
Subcostal outflow tract view The red arrow shows the pulmonary artery originating from the left ventricle. LV, left ventricle; RV, right ventricle; TV, tricuspid valve; PV, pulmonary valve; MPA, main pulmonary artery; LPA, left pulmonary artery; RPA, right pulmonary artery

Lastly, the subcostal outflow tract view corresponding to Figure 3 was obtained from below the ribcage, allowing visualization of the heart and significant vasculature from a different angle. It is very commonly used in pediatric cases. In this picture, the pulmonary artery originates more posteriorly from the left side of the heart, along with its bifurcation. The aorta is noticed to originate more anteriorly and from the right side. The VSD can also be demonstrated and profiled from this view.

Management

In newborns as young as one month old, this condition necessitates a comprehensive approach encompassing both medical management and surgical intervention. The initial goal of management focuses on stabilizing the child's condition through comprehensive medical care, which aims to optimize the tissue oxygenation and alleviate symptoms of heart failure, and subsequently prepare and make the child fit for surgical intervention. Once the child is stabilized, surgical intervention can be done in the form of an Arterial Switch Operation (ASO), which remains the definitive treatment of TGA.

Medical Management

Medical management of TGA mainly focuses on stabilizing the child's condition while making the child fit to undergo a surgical repair. The following is the approach taken to manage the child medically when he first presented. The patient was stabilized by ensuring adequate tissue oxygenation through continuous positive airway pressure (CPAP). Hydration and the maintenance of cardiac output were done by giving intravenous fluids. Injection of cefotaxime was given intravenously as a prophylaxis to infectious agents like opportunistic bacteria. To counteract the underlying hypokalemia, potassium chloride was administered. To retain the patency of the ductus arteriosus, prostaglandin E1 was administered. Diuretics like furosemide were used to reduce fluid overload and reduce the symptoms of heart failure by promoting diuresis. A pediatric cardiologist also performed an extensive preoperative evaluation to determine the following approach.

Surgical Management

Surgical restoration is the only mainstay of definitive therapy in the case of TGA, which recreates a very critical part of the survival of the child. The most widely accepted and successful surgery performed in the cases of TGA is an arterial switch operation (ASO) along with the closure of the VSD. The following are the steps that were carried out during the surgery.

Surgery notes

The infant was operated on after acquiring informed consent from the parents, along with anesthetic clearance. The precordium was cleaned and draped after putting a sandbag under the neck. A median sternotomy was done to access the heart and the great vessels. The patient then was put on cardiopulmonary bypass (CPB) to divert the blood flow distal to the heart, allowing a bloodless surgical field. Cardiac arrest is acquired by infusion of a cardioplegic solution into the coronaries, allowing the heart to be still during surgery. Mobilization and transection of the aorta and pulmonary artery were slightly higher than the respective placement at valve levels. Translocation of the coronary arteries to the neo-aorta and reconstruction of the pulmonary artery was done. The closure of the VSD was done using a Dacron patch of appropriate size. Weaning off CPB was done to restore the circulation gradually and close the sternum and surgical incision. The infant was then moved to the Pediatric Intensive Care Unit (PICU). The postoperative period was uneventful, but the patient tolerated the procedure well. Continuous assessment of cardiac function, oxygenation, and perfusion was done. Gradual weaning from mechanical ventilation and stabilization of vital signs was carried out. Initiation of enteral feeds as breastfeeding was started. Long-term follow-up with a pediatric cardiologist was advised.

## Discussion

The following congenital malformation is encountered in infants and is not very common. It causes cyanotic abnormalities even when the ventricular septum is not involved [8]. The circulatory mixing due to the patency of the ductus arteriosus is seen to have a paradoxical positive outcome in the child. Therefore, its physiological closure may lead to acute cyanosis and clinical deterioration. Newborns presenting with cyanosis can be managed via a percutaneous procedure, in which an atrial balloon septostomy is done to generate a significant defect in the atrial septum, which could significantly enhance tissue oxygenation [9]. In this condition, the structurally normal right atrium is linked to the structural right ventricle, from which the entire or majority of the aorta arises.

By contrast, the structurally normal left atrium is correlated to the structural left ventricle, from which the pulmonary trunk originates [10]. With the introduction of newer and technologically advanced procedures, along with better postoperative maintenance, the surveillance of this condition is successful in most cases, with augmented long-term survival rates [11]. Nonetheless, latent research has found a compromise in exercise performance, decreased accommodation of cognitive functioning, and a poor quality of life [12].

## Conclusions

This case report accentuates the critical presentation of a one-month-old infant diagnosed with transposition of great arteries accompanied by a 6 mm perimembranous VSD. Irrespective of the challenges faced in this complex congenital heart defect, early diagnosis and appropriate medical management execute a very climactic function in improving the prognosis. This case report accentuates the significance of a multidisciplinary technique and continuous monitoring in managing such cases. It emphasizes the need for extensive research and advancements in pediatric cardiology to improve outcomes for infants with similar congenital defects. Furthermore, the diagnostic modalities, therapeutic strategies, and surgical techniques must be developed even more. This can be achieved by continuously expanding the understanding and scope of congenital cardiac deformities and refining our approach to treatment. Thus, we can strive towards better outcomes and improved quality of life for infants with complex cardiac defects. This case report also serves as a poignant reminder of the resilience of young patients and the profound impact of multidisciplinary care in mitigating the challenges posed by congenital heart diseases.
